# Simultaneous Changes of Spatial Memory and Spine Density after Intrahippocampal Administration of Fibrillar A*β*_1–42_ to the Rat Brain

**DOI:** 10.1155/2014/345305

**Published:** 2014-06-23

**Authors:** Emőke Borbély, János Horváth, Szabina Furdan, Zsolt Bozsó, Botond Penke, Lívia Fülöp

**Affiliations:** Department of Medical Chemistry, University of Szeged, Dóm tér 8, Szeged 6720, Hungary

## Abstract

Several animal models of Alzheimer's disease have been used in laboratory experiments. Intrahippocampal injection of fibrillar amyloid-beta (fA*β*) peptide represents one of the most frequently used models, mimicking A*β* deposits in the brain. In our experiment synthetic fA*β*_1–42_ peptide was administered to rat hippocampus. The effect of the A*β* peptide on spatial memory and dendritic spine density was studied. The fA*β*_1–42_-treated rats showed decreased spatial learning ability measured in Morris water maze (MWM). Simultaneously, fA*β*_1–42_ caused a significant reduction of the dendritic spine density in the rat hippocampus CA1 region. The decrease of learning ability and the loss of spine density were in good correlation. Our results prove that both methods (MWM and dendritic spine density measurement) are suitable for studying A*β*-triggered neurodegeneration processes.

## 1. Introduction

Alzheimer's disease (AD) is a progressive neurodegenerative disorder characterized by deficit of learning process, severe memory loss, and complex behavioural changes [1–5]. Neuropathological hallmarks of AD include the cerebral accumulation of extracellular senile plaques containing various forms of amyloid-beta (A*β*) peptide assemblies and the presence of intracellular neurofibrillary tangles containing tau protein [6–8]. Other features of AD are neuroinflammation, cerebrovascular alterations, activated astrocytes, and microglia as well as synaptic and neuronal loss in specific brain regions [9–11]. The affected brain regions are forebrain and medial temporal lobe structures like the hippocampus (HC), the entorhinal cortex, and the amygdala [11]. Synapse loss is strongly correlated with cognitive impairment; thus synapse number is the best indicator of cognitive decline in AD [1, 12]. The senile plaques are associated with local synapse and dendritic spine loss [13, 14]. Moreover, the fibrillar deposits are surrounded by a halo of oligomeric A*β* assemblies [15]. Extracellular oligomeric A*β* associates with dendritic spines covering their surface [15]. In addition, intracellular A*β* may also contribute to AD pathology by tau hyperphosphorylation and synaptic dysfunction [16]. The toxic effects of different aggregation forms of A*β* (oligomers, protofibrils, and fibrils) have not been revealed completely yet.

Dendritic spines are cellular compartments containing the molecular machinery important for synaptic transmission and plasticity [17]. In pyramidal neurons of the hippocampus, there is an almost one-to-one relationship between the number of dendritic spines and excitatory synapses [17, 18]. The loss of dendritic spines and the presence of dystrophic neurites have been reported both in the amyloid precursor protein (APP) overexpressing transgenic mouse model of AD and in the AD-affected human brain [2, 8, 19, 20]. The spine density of prefrontal cortex neurons is greatly reduced in aged monkeys as a hallmark of cognitive decline [21]. Neurocortical pyramidal neurons have extensive apical and basilar dendritic trees to integrate information from excitatory and inhibitory synaptic inputs. In the neuronal network, dendritic spines represent the principal receptive sites for the excitatory inputs to the neurons. The strength, stability, and function of the excitatory synaptic connections constitute the basis of cognitive function [22]. In the cerebral cortex of mammals, the rapid synaptogenesis during early postnatal life is followed by a substantial loss of synapses/spines that extends through adolescence. In adulthood, the number of spines remains relatively stable and then decreases progressively with aging [22, 23]. Spines appear and disappear through life, but their turnover rate declines with the age and is regulated by the neuronal activity [18]. Dendritic spines undergo structural modifications when the synaptic strength is experimentally modified (e.g., by evoking long-term potentiation or long-term depression) [18, 22, 24].

Extracellular delivery of A*β* peptides can initiate synaptic loss in rodent brains [25]. Intrahippocampal (IHC) administration of A*β* is a widely used animal model to study AD [4, 25–38]. The injection of A*β* into murine brain rapidly establishes the symptoms of AD. This method is suitable for studying the effects of the different aggregates of A*β*. There are conflicting results about the neurotoxicity of *β*-amyloid plaque depositions [39]; however, the toxic effect of A*β* peptides on synapses has been widely acknowledged.

Reduction of dendritic spine density has been shown in cell cultures after A*β* treatment [40–43]. Furthermore, IHC administration of fibrillar A*β*_1–40_ has been shown to decrease spine density [25, 28]. Interestingly, no experiments have been performed using well characterized A*β*_1–42_, the most toxic form of A*β* peptides. Thus the aim of this study was to assess the impact of fibrillar A*β*_1–42_ (fA*β*_1–42_) administration on spatial memory of rats and dendritic spine density in the hippocampus. Our hypothesis was that IHC injected synthetic, fibrillar form of A*β*_1–42_ could simultaneously influence and reduce the learning process and dendritic spine density. Since hippocampal dendritic spines are the key elements in acquisition and retention and have been implicated in learning and memory processes [44, 45], we also compared the correlation between the changes of dendritic spine density (induced by fA*β*_1–42_ administration) and the spatial memory of rats.

## 2. Materials and Methods

### 2.1. Animals

Male Charles-River Wistar rats (*n* = 24), weighing about 210–230 g at the beginning of the experiment, were used as subjects. Two groups, control (*n* = 12) and fA*β*_1–42_-treated (*n* = 12), were formed. They were housed in groups of three under constant temperature, humidity, and lighting conditions (23°C, 12 : 12 h light/dark cycle, lights on at 7 a.m.). Standard rat chow and tap water were supplied* ad libitum*. All behavioural procedures were conducted during the light phase. Handling was done daily at the same time. Experiments were performed in accordance with the European Communities Council Directive of 22 September 2010 (2010/63/EU on the protection of animals used for scientific purposes). Formal approval to conduct the experiments was obtained from the Animal Experimentation Committee of the University of Szeged.

### 2.2. Synthesis of A*β*_1–42_ and Preparation of the Fibrillar Peptide Aggregates

**Figure 1:**
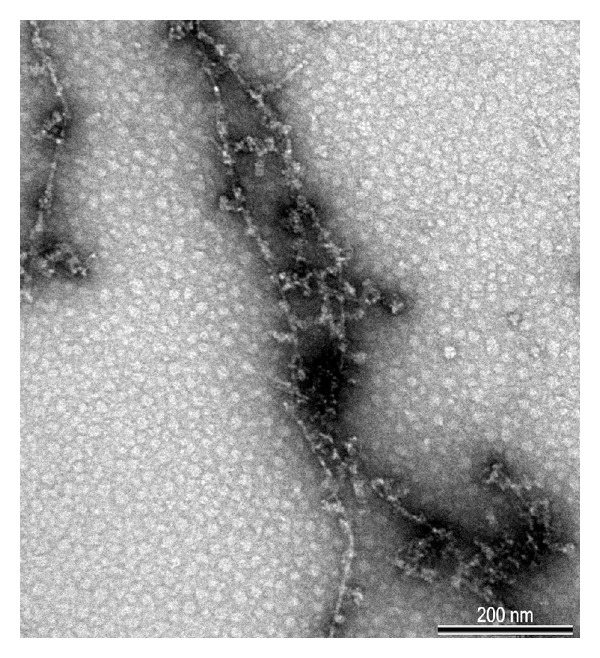
Representative electron microscope photomicrograph of the injected amyloid-beta fibrils.

The synthesis was performed as reported earlier in Bozso et al. [46]. The fA*β*_1–42_ was prepared as described by He et al. [4]. Briefly, A*β*_1–42_ was dissolved in hexafluoroisopropanol (HFIP, Sigma Aldrich) to 1 mM; HFIP was removed* in vacuo*. The peptide was suspended to make a 5 mM solution in dimethyl sulfoxide (DMSO, Sigma Aldrich). The fibrillar form was prepared diluting the DMSO stock solution of the peptide with a 100 mM HEPES buffer to a final concentration of 222 *μ*M. The solution was incubated at 37°C for 7 days. After the aggregation period the sample was centrifuged for 10 min at 15000 g at room temperature. The pellet containing the freshly prepared fibrillar A*β*_1–42_ was resuspended in 100 mM HEPES buffer (pH 7.5) and used in the experiment. The samples containing A*β* fibrils were characterized by transmission electron microscope (Philips CM10, FEI, Eindhoven, Figure 1).

### 2.3. Surgery

Rats were anaesthetized with an intraperitoneal injection of a mixture of ketamine (10.0 mg/0.1 kg) and xylasine (0.8 mg/0.1 kg). The animals were then placed in a stereotaxic apparatus, a midline incision of the scalp was made, the skin and muscles were carefully retracted to expose the skull, and a hole was drilled above the target area. The solution was injected with a Hamilton syringe into the right HC unilaterally at a rate of 1.0 *μ*L/min, beginning 2 min after the needle was lowered. The needle was removed very slowly 2 min after the end of injection. The following coordinates were used (from Bregma point): AP: −3.6; ML: −2.4; DV: −2.8 [47]. Rats were randomly injected either with the fibrillar form of A*β*_1–42_ (222 *μ*M A*β*_1–42_) or with vehiculum (physiological saline). The A*β*_1–42_-injected and the control animals were treated with antibiotics and analgesics after the surgery.

### 2.4. Spatial Navigation in the Morris Water Maze

Spatial learning and memory were assessed in a Morris water maze (MWM) on days 14 to 20 after IHC fA*β*_1–42_ administration. Behavioural testing was carried out in a room illuminated by three lamps giving diffuse light of approximately equal intensity at all points of the maze. The maze consisted of a circular pool (*d* = 180 cm, *h* = 60 cm) filled with water (23 ± 1°C) and made opaque with milk. A black curtain was positioned around the pool with distal cues. A video camera was mounted on the ceiling directly above the test apparatus and relied to a video tracking system. The behaviour of the animals was automatically recorded with the software EthoVision 2.3 (Noldus Information Technology, The Netherlands, 2002).

Memory acquisition trials (training period) were performed daily during the light phase, in blocks of 4, for 6 days [4]. The pool was divided into four virtual quadrants, and an invisible escape platform (diameter: 10 cm) was submerged in the middle of one of the four quadrants 1.5 cm below the water surface. At each trial, the rats were allowed to swim freely for a maximum of 120 sec until they found the platform and were then allowed to stay on it for 10 sec. If the rat failed to find the platform within 120 sec, it was guided to or was placed on the island manually for 10 sec. During the acquisition period, four different starting points were used and the starting positions were varied pseudorandomly over the trials. Twenty-four hours after the last acquisition trial, retention was assessed in a 120 sec probe trial, with the platform removed.

The data recorded by video tracking were used to calculate the time to reach the platform, swim speed, and swim path length (distance) during acquisition trials as well as percent time spent in each of the 4 virtual quadrants and time spent and number of crossings over the platform's position during the probe test.

### 2.5. Quantification of Dendritic Spine Density Using Golgi Impregnation

FD Rapid GolgiStain Kit (FD NeuroTechnologies, Consulting & Services, Inc., USA) was used according to the manufacturers' instructions. Rats (*n* = 6, 3 per group) were deeply anesthetized before the brain was removed from the skull. The sacrificing was made after 29 days of the IHC injection. The brains were removed quickly and handled carefully to avoid damage of the tissue, and then tissue blocks including hippocampus (approximately 0.7-0.8 cm) were cut from the brain. The tissue blocks were immersed in the impregnation solution (A + B solution) and stored at room temperature for 2 weeks in the dark. After the first impregnation period the brains were transferred into the second solution (C) and stored at 4°C in the dark for at least 48 hours.

100 *μ*m coronal sections were cut with a vibration microtome (Zeiss Microm HM 650 V) and were mounted on gelatin coated glass slides. After the staining procedure (D, E solution) and dehydration, the slides were coverslipped with DPX mountant for histology (VWR International).

### 2.6. Quantitative Analysis

The Golgi sections were studied by inverse light microscopy, using oil immersion objectives. A total of 25 pyramidal neurons from the dorsoventral hippocampal CA1 (stratum radiatum) were studied from each of the 6 animals (75 dendritic shafts per group were analyzed). The spine density of the proximal apical dendrite area was analyzed (minimum 100 *μ*m from soma). For each examined neuron, one 100 *μ*m long segment from a second- or third-order dendrite (protruding from its parent apical dendrite) was chosen for spine density quantification as previously described [48]. The dendrites were selected under a 100x oil immersion lens and the images of these apical dendrites were captured through a camera (AxioCam MRC V5, program: AxioVision 40 V. 4.8.1.0 Carl Zeiss Imaging Solutions GmbH) connected to a light microscope (Zeiss Observer Z1, with 10x ocular magnification) and a computer. Serial images were made from each dendrite in the whole of the analyzed segment (Z-stack). The captured multiple photomicrographs from one dendrite were then stacked into one file. To stack the images and determine the spine density, ImageJ 1.44 software (National Institute of Health, Bethesda, USA) was used.

### 2.7. Statistical Analysis

Behavioural data were analyzed by repeated measures ANOVA, followed by Fisher's LSD post hoc tests for multiple comparisons. For the evaluation of the results of Golgi impregnation, Student's *t*-test for independent samples was used. Statistical significance was set at *P* < 0.05. The data were expressed as the means ± (S.E.M.).

## 3. Results

### 3.1. Spatial Learning and Memory in the Morris Water Maze

**Figure 2:**
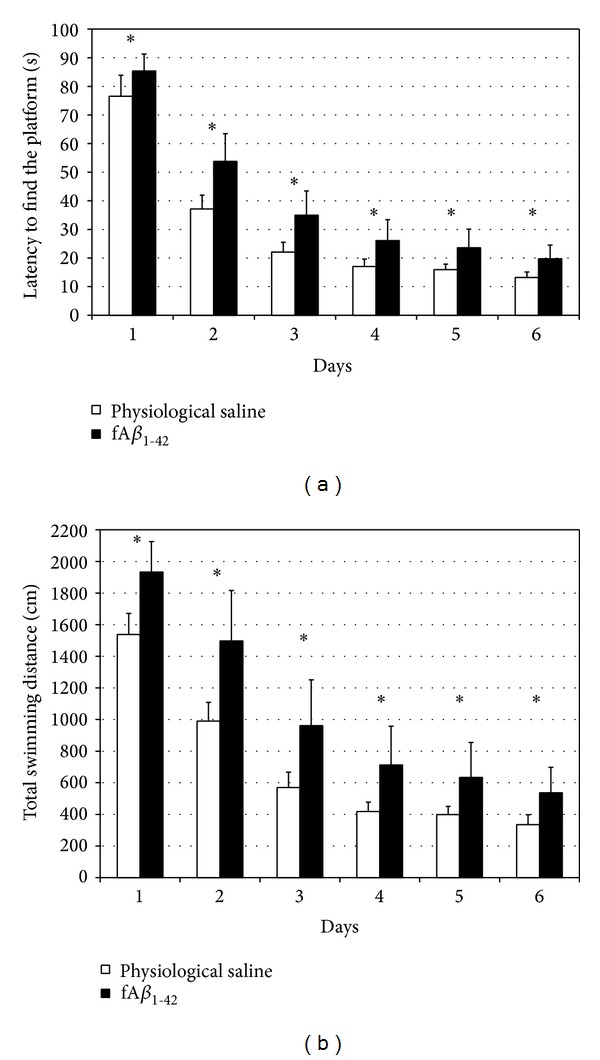
fA*β*_1–42_-injection induced learning deficit in the Morris water maze test. (a) fA*β*_1–42_-injection resulted in slower learning during the acquisition phase compared to control group (*P* = 0.013); (b) fA*β*_1–42_-treated animals swam longer distance than the controls to find the platform (*P* = 0.010). Each value represents the mean (±S.E.M.) (*n* = 12 rats per group).

MWM was used to test spatial learning and memory each day on days 14 to 20 after IHC administration of fA*β*_1–42_. Escape latency to find the platform was used as a measure for evaluating spatial memory. The results showed that the performance of both groups (fA*β*-treated and untreated) improved from day to day, reflecting long-term memory. However, learning was slower each day in the fA*β*_1–42_-treated compared to the control group: escape latencies were significantly longer in rats with fA*β*_1–42_ treatment than in the control animals analysed by repeated measures ANOVA (*F*_1,94_ = 6.450; *P* = 0.013) (Figure 2(a)). A significant difference was observed between the groups also for swimming distance (repeated measures ANOVA: *F*_1,94_ = 6.840; *P* = 0.010) (Figure 2(b)).

**Figure 3:**
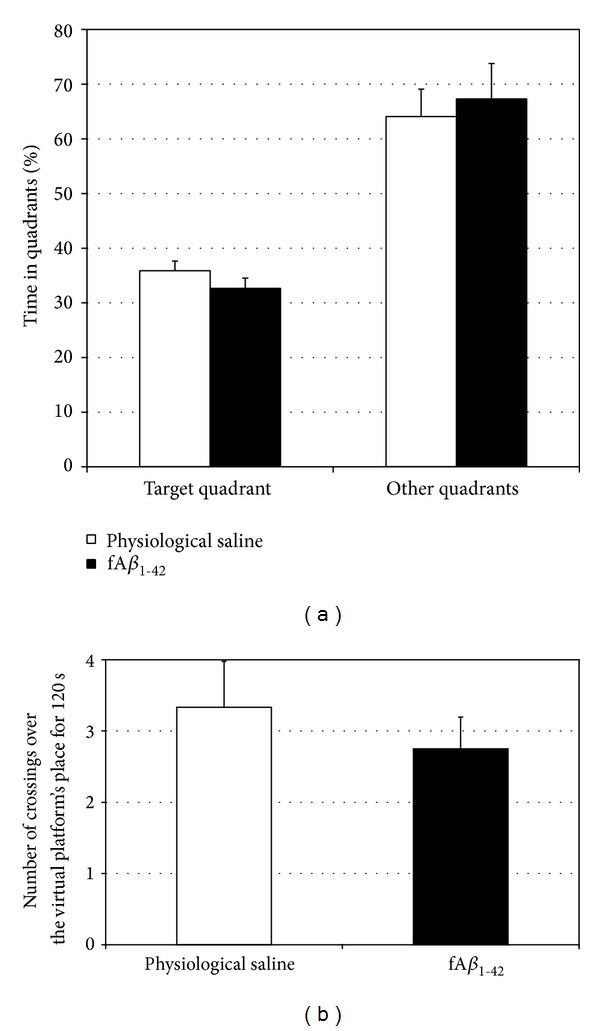
Injection of fA*β*_1–42_ did not affect performance in the Morris water maze probe test. (a) In the target quadrant, the amyloid-treated animals spent time comparable to controls. (b) fA*β*_1–42_-injection did not have a significant effect on the number of crossings over where the virtual platform had been in the maze. Each value represents the mean (±S.E.M.) (*n* = 12 rats per group).

Despite the significantly slower learning in the fA*β*_1–42_-treated group, performance at the probe test (given 24 h after the learning phase) indicated that spatial memory was not impaired, as the time spent in quadrants and the number of crossings over the virtual platform's position were comparable in the two groups (*t*_22_ = −1.247; *P* = 0.226 and *t*_22_ = 0.745; *P* = 0.464, resp.) (Figures 3(a) and 3(b)).

### 3.2. Dendritic Spine Density

The Golgi staining method labelled a subset of neurons in the hippocampus. In general, 20–30 fully impregnated CA1 pyramidal cells could be detected per slice. There was no difference between the groups in staining.

**Figure 4:**
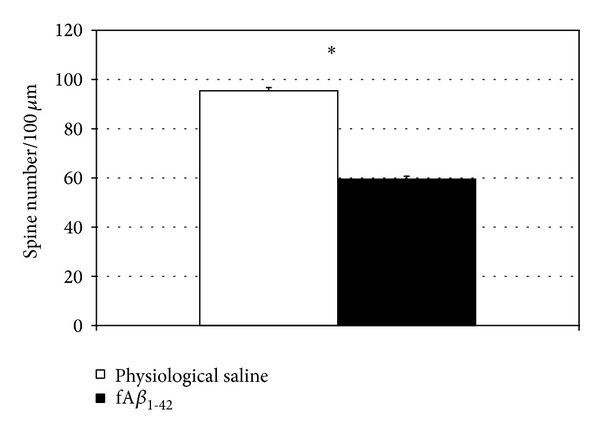
Golgi staining revealed changes in spine density after the fA*β*_1–42_-injection (A). Apical dendritic spine density analysis showed that the amyloid treatment induced a decrease in spine density (*P* < 0.0001). In each experimental group 75 dendritic shafts of 3 animals were studied. The values represent the mean (±S.E.M.) (*n* = 3 rats per group).

We investigated all types of spines but solely focused on determining dendritic spine density. Spine numerical density was different between the two groups (*t*_28_ = 14.415; *P* < 0.0001). In the fA*β*_1–42_-treated group decreasing spine density (spine number) was detected compared to the controls (Figure 4). Each column represents the mean value of the dendritic spine number of 75-75 pyramidal neurons.

**Figure 5:**
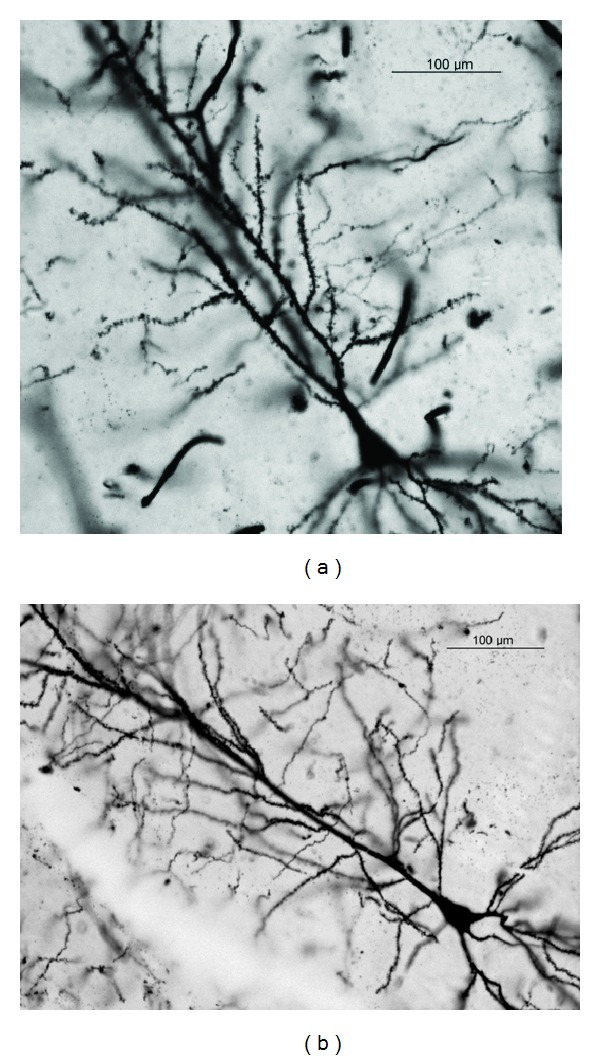
Representative photomicrograph of a CA1 subfield pyramidal neuron from a control (a) and an amyloid-treated (b) rat.

**Figure 6:**
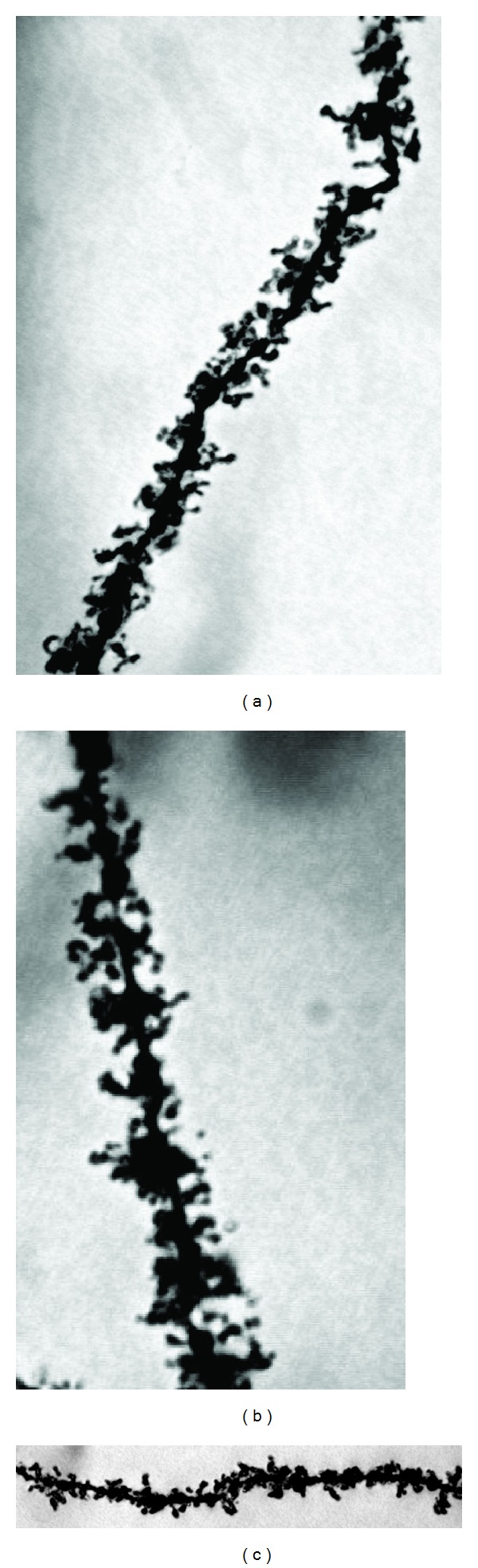
Representative photomicrographs of oblique dendritic segments from hippocampal CA1 pyramidal neurons of three control rats ((a), (b), and (c)). The dendritic spine density of vehiculum treated animals was significantly higher compared to the fA*β*_1–42_-treated rats. (1000x).

**Figure 7:**
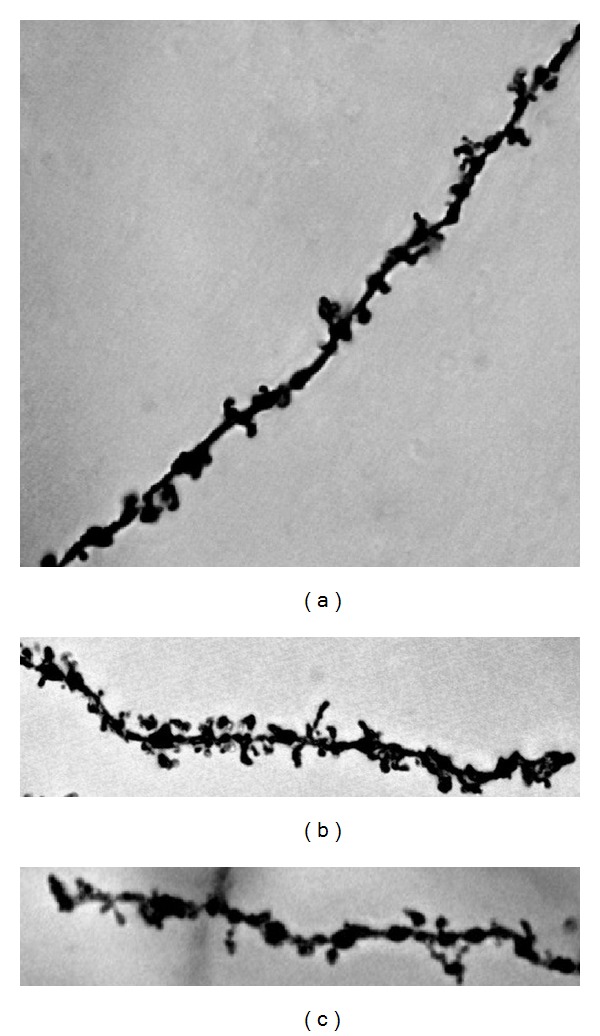
Representative photomicrographs of oblique dendritic segments from hippocampal CA1 pyramidal neurons of every amyloid-treated Golgi impregnated rat ((a), (b), and (c)). The fA*β*_1–42_ locally reduced the spine density in the fA*β*_1–42_-injected group (1000x).

Photomicrographs of the pyramidal neurons from control, nontreated rats (Figures 5(a) and 6), and fA*β*_1–42_-treated rats (Figures 5(b) and 7) clearly demonstrate the difference in dendritic spine density in the two experimental groups.

## 4. Discussion

The present study explored the effects of fA*β*_1–42_ on spatial behaviour and on hippocampal dendritic spines in nontransgenic rats. A*β* accumulation in the specific brain regions is a hallmark of AD pathology; however, the role of amyloid plaques has been debated. Depositions of fA*β* in plaques and the surrounding oligomeric A*β* are considered as trigger signals to induce dendritic spine loss and synapse dysfunction in AD. A*β* assemblies are synaptotoxic: they can be bound to axons and membrane proteins, resulting in Ca^2+^ influx into the neurons [49]. The synapse and dendritic spine loss are strongly correlated with cognitive impairment in AD, and A*β* has been shown to target synapses [40, 50].

Numerous studies have reported significant changes after IHC administration of various types and aggregation forms of A*β* [4, 25–38]. Some studies reported that synapse dysfunction was triggered by A*β* oligomers [49, 51]. Other studies proposed fA*β* deposits as causative factor for the local synaptic abnormalities since decrease of dendritic spine density was detected nearby the A*β* plaques [13, 52–54]. However, it has not been clear how the nondiffusible, immobile A*β* fibrils interact with neuronal structure. As of today, senile plaques are considered mostly as nontoxic “outburns” sequestering toxic A*β* species to nontoxic fibrils. However, bilateral IHC injection of fA*β*_1–42_ results in reduction of neuronal density and increases of glial fibrillary acidic protein intensity, with simultaneous appearance of numerous A*β* deposits and behavioural performance deficits [4, 26]. IHC administration of shorter form of A*β* (A*β*_1–40_) results in decreased density of dendritic spines in hippocampus [25, 28].

Our current findings demonstrated that synthetic fA*β*_1–42_ simultaneously decreased spatial learning ability measured in MWM (Figure 2(a)) and reduced dendritic spine density in the rat hippocampus CA1 region (Figures 4–6). According to the literature data, the synthetic fA*β* assemblies have also a surrounding of A*β* oligomers [55], in accordance with the law of chemical equilibriums. After fA*β* injection, diffusible A*β* oligomers could be formed in the rat hippocampus and initiate dendritic spine density loss. The measured spine loss in HC CA1 region may explain the decreased learning ability since the presence and maturation of dendritic spines on the CA1 pyramidal cells are necessary to evolve the spatial memory unit [56]. It is generally accepted that misfolded proteins initiate dendritic spine reduction and memory decline. A*β* and *α*-synuclein oligomers decrease the amount of synaptic proteins and vesicles and via tau hyperphosphorylation initiate the loss of dendritic spines [57, 58]. The instability of dendritic spines leads to progressive neocortical spine loss in a mouse model of Huntington's disease [59]. Our results support the theory that decreasing spine density in AD can cause or contribute to the memory decline: dendritic spines are the site of most excitatory synapses and their loss is in good correlation with the cognitive dysfunction.

## Abbreviations

AD: Alzheimer's disease
APP: Amyloid precursor protein
A*β*: Amyloid-beta
DMSO: Dimethyl sulfoxide
fA*β*_142_: Fibrillar A*β*_142_
HC: Hippocampus
HFIP: Hexafluoroisopropanol
IHC: Intrahippocampal
MWM: Morris water maze.

## References

[1] Terry RD, Masliah E, Salmon DP (1991). Physical basis of cognitive alterations in Alzheimer’s disease: synapse loss is the major correlate of cognitive impairment. *Annals of Neurology*.

[2] Allan Butterfield D, Castegna A, Lauderback CM, Drake J (2002). Evidence that amyloid beta-peptide-induced lipid peroxidation and its sequelae in Alzheimer’s disease brain contribute to neuronal death. *Neurobiology of Aging*.

[3] Kar S, Slowikowski SPM, Westaway D, Mount HTJ (2004). Interactions between *β*-amyloid and central cholinergic neurons: implications for Alzheimer’s disease. *Journal of Psychiatry and Neuroscience*.

[4] He F-Q, Qiu B-Y, Zhang X-H (2011). Tetrandrine attenuates spatial memory impairment and hippocampal neuroinflammation via inhibiting NF-*κ*B activation in a rat model of Alzheimer’s disease induced by amyloid-*β*
_1-42_. *Brain Research*.

[5] Pause BM, Zlomuzica A, Kinugawa K, Mariani J, Pietrowsky R, Dere E (2013). Perspectives on episodic-like and episodic memory. *Frontiers in Behavioral Neuroscience*.

[6] Braak H, Braak E (1991). Neuropathological stageing of Alzheimer-related changes. *Acta Neuropathologica*.

[7] Butterfield DA, Perluigi M, Sultana R (2006). Oxidative stress in Alzheimer’s disease brain: new insights from redox proteomics. *European Journal of Pharmacology*.

[8] Baloyannis SJ (2009). Dendritic pathology in Alzheimer’s disease. *Journal of the Neurological Sciences*.

[9] Selkoe DJ (1999). Translating cell biology into therapeutic advances in Alzheimer’s disease. *Nature*.

[10] Selkoe DJ (2001). Alzheimer’s disease results from the cerebral accumulation and cytotoxicity of amyloid *β*-protein. *Journal of Alzheimer’s Disease*.

[11] Mattson MP (2004). Pathways towards and away from Alzheimer’s disease. *Nature*.

[12] Chabrier MA, Cheng D, Castello NA, Green KN, Laferla FM (2014). Synergistic effects of amyloid-beta and wild-type human tau on dendritic spine loss in a floxed double transgenic model of Alzheimer's disease. *Neurobiology of Disease*.

[13] Tsai J, Grutzendler J, Duff K, Gan W-B (2004). Fibrillar amyloid deposition leads to local synaptic abnormalities and breakage of neuronal branches. *Nature Neuroscience*.

[14] Knafo S, Alonso-Nanclares L, Gonzalez-Soriano J (2009). Widespread changes in dendritic spines in a model of Alzheimer’s Disease. *Cerebral Cortex*.

[15] Koffie RM, Meyer-Luehmann M, Hashimoto T (2009). Oligomeric amyloid *β* associates with postsynaptic densities and correlates with excitatory synapse loss near senile plaques. *Proceedings of the National Academy of Sciences of the United States of America*.

[16] LaFerla FM, Green KN, Oddo S (2007). Intracellular amyloid-*β* in Alzheimer’s disease. *Nature Reviews Neuroscience*.

[17] Nimchinsky EA, Sabatini BL, Svoboda K (2002). Structure and function of dendritic spines. *Annual Review of Physiology*.

[18] Alvarez VA, Sabatini BL (2007). Anatomical and physiological plasticity of dendritic spines. *Annual Review of Neuroscience*.

[19] Smith DL, Pozueta J, Gong B, Arancio O, Shelanski M (2009). Reversal of long-term dendritic spine alterations in Alzheimer disease models. *Proceedings of the National Academy of Sciences of the United States of America*.

[20] Jain S, Yoon SY, Leung L, Knoferle J, Huang Y (2013). Cellular source-specific effects of apolipoprotein (apo) E4 on dendrite arborization and dendritic spine development. *PLoS ONE*.

[21] Morrison JH, Baxter MG (2012). The ageing cortical synapse: Hallmarks and implications for cognitive decline. *Nature Reviews Neuroscience*.

[22] Luebke JI, Weaver CM, Rocher AB (2010). Dendritic vulnerability in neurodegenerative disease: insights from analyses of cortical pyramidal neurons in transgenic mouse models. *Brain Structure and Function*.

[23] Bhatt DH, Zhang S, Gan W-B (2009). Dendritic spine dynamics. *Annual Review of Physiology*.

[24] Matsuzaki M, Honkura N, Ellis-Davies GCR, Kasai H (2004). Structural basis of long-term potentiation in single dendritic spines. *Nature*.

[25] Wan B, Hu X, Nie J (2013). Effects of triptolide on degeneration of dendritic spines induced by A*β*
_1-40_ injection in rat hippocampus. *Neurological Sciences*.

[26] Chacón MA, Barría MI, Soto C, Inestrosa NC (2004). *β*-sheet breaker peptide prevents A*β*-induced spatial memory impairments with partial reduction of amyloid deposits. *Molecular Psychiatry*.

[27] Zhong S-Z, Ge Q-H, Li Q, Qu R, Ma S-P (2009). Peoniflorin attentuates A*β*
_1-42_-mediated neurotoxicity by regulating calcium homeostasis and ameliorating oxidative stress in hippocampus of rats. *Journal of the Neurological Sciences*.

[28] Gong X, Lu X, Zhan L (2010). Role of the SNK-SPAR pathway in the development of Alzheimer’s disease. *IUBMB Life*.

[29] Li J, Wang G, Liu J (2010). Puerarin attenuates amyloid-beta-induced cognitive impairment through suppression of apoptosis in rat hippocampus in vivo. *European Journal of Pharmacology*.

[30] Choi JG, Moon M, Kim HG (2011). Gami-Chunghyuldan ameliorates memory impairment and neurodegeneration induced by intrahippocampal A*β*
_1-42_ oligomer injection. *Neurobiology of Learning and Memory*.

[31] Jantaratnotai N, Ryu JK, Schwab C, McGeer PL, McLarnon JG (2011). Comparison of vascular perturbations in an A*β*-injected animal model and in AD brain. *International Journal of Alzheimer’s Disease*.

[32] Quan Q, Wang J, Li X, Wang Y (2013). Ginsenoside Rg1 decreases A*β*
_1-42_ level by upregulating PPAR*γ* and IDE expression in the hippocampus of a rat model of Alzheimer’s disease. *PLoS ONE*.

[33] He P, Ouyang X, Zhou S (2013). A novel melatonin agonist Neu-P11 facilitates memory performance and improves cognitive impairment in a rat model of Alzheimer’ disease. *Hormones and Behavior*.

[34] Liu X-J, Yuan L, Yang D (2013). Melatonin protects against amyloid-*β*-induced impairments of hippocampal LTP and spatial learning in rats. *Synapse*.

[35] Zheng M, Liu J, Ruan Z (2013). Intrahippocampal injection of A*β*
_1-42_ inhibits neurogenesis and down-regulates IFN-*γ* and NF-*κ*B expression in hippocampus of adult mouse brain. *Amyloid*.

[36] Jia J, Kang L, Li S (2013). Amelioratory effects of testosterone treatment on cognitive performance deficits induced by soluble Abeta_1-42_ oligomers injected into the hippocampus. *Hormones and Behavior*.

[37] Yin Y, Ren Y, Wu W (2013). Protective effects of bilobalide on A*β*
_25-35_ induced learning and memory impairments in male rats. *Pharmacology Biochemistry and Behavior*.

[38] Zhang J, Zhena Y-F, Song L-G (2013). Salidroside attenuates beta amyloid-induced cognitive deficits via modulating oxidative stress and inflammatory mediators in rat hippocampus. *Behavioural Brain Research*.

[39] Bishop GM, Robinson SR (2002). The amyloid hypothesis: let sleeping dogmas lie?. *Neurobiology of Aging*.

[40] Wei W, Nguyen LN, Kessels HW, Hagiwara H, Sisodia S, Malinow R (2010). Amyloid beta from axons and dendrites reduces local spine number and plasticity. *Nature Neuroscience*.

[41] Chang PK-Y, Boridy S, McKinney RA, Maysinger D (2013). Letrozole potentiates mitochondrial and dendritic spine impairments induced by *β* amyloid. *Journal of Aging Research*.

[42] Meng C, He Z, Xing D (2013). Low-level laser therapy rescues dendrite atrophy via upregulating BDNF expression: implications for Alzheimer’s disease. *Journal of Neuroscience*.

[43] Nath S, Agholme L, Kurudenkandy FR, Granseth B, Marcusson J, Hallbeck M (2012). Spreading of neurodegenerative pathology via neuron-to-neuron transmission of *β*-amyloid. *Journal of Neuroscience*.

[44] Leuner B, Mendolia-Loffredo S, Kozorovitskiy Y, Samburg D, Gould E, Shors TJ (2004). Learning enhances the survival of new neurons beyond the time when the hippocampus is required for memory. *Journal of Neuroscience*.

[45] Leuner B, Shors TJ (2004). New spines, new memories. *Molecular Neurobiology*.

[46] Bozso Z, Penke B, Simon D (2010). Controlled in situ preparation of A*β*
_1-42_ oligomers from the isopeptide “iso-A*β*
_1-42_”, physicochemical and biological characterization. *Peptides*.

[47] Paxinos G, Watson C (1982). *The Rat Brain in Stereotaxic Coordinates*.

[48] Nagy D, Kocsis K, Fuzik J (2011). Kainate postconditioning restores LTP in ischemic hippocampal CA1: onset-dependent second pathophysiological stress. *Neuropharmacology*.

[49] Selkoe DJ (2002). Alzheimer’s disease is a synaptic failure. *Science*.

[50] Lacor PN, Buniel MC, Chang L (2004). Synaptic targeting by Alzheimer’s-related amyloid *β* oligomers. *Journal of Neuroscience*.

[51] Lacor PN, Buniel MC, Furlow PW (2007). A*β* oligomer-induced aberrations in synapse composition, shape, and density provide a molecular basis for loss of connectivity in Alzheimer’s disease. *Journal of Neuroscience*.

[52] Grutzendler J, Helmin K, Tsai J, Gan W-B (2007). Various dendritic abnormalities are associated with fibrillar amyloid deposits in Alzheimer’s disease. *Annals of the New York Academy of Sciences*.

[53] Kirkwood CM, Ciuchta J, Ikonomovic MD (2013). Dendritic spine density, morphology, and fibrillar actin content surrounding amyloid-*β* plaques in a mouse model of amyloid-*β* deposition. *Journal of Neuropathology and Experimental Neurology*.

[54] Le R, Cruz L, Urbanc B (2001). Plaque-induced abnormalities in neurite geometry in transgenic models of Alzheimer disease: implications for neural system disruption. *Journal of Neuropathology and Experimental Neurology*.

[55] Sandberg A, Luheshi LM, Sollvander S (2010). Stabilization of neurotoxic Alzheimer amyloid-beta oligomers by protein engineering. *Proceedings of the National Academy of Sciences of the United States of America*.

[56] Feria-Velasco A, del Angel AR, Gonzalez-Burgos I (2002). Modification of dendritic development. *Progress in Brain Research*.

[57] Kramer ML, Schulz-Schaeffer WJ (2007). Presynaptic *α*-synuclein aggregates, not Lewy bodies, cause neurodegeneration in dementia with lewy bodies. *Journal of Neuroscience*.

[58] Overk CR, Masliah E (2014). Pathogenesis of synaptic degeneration in Alzheimer's disease and Lewy body disease. *Biochemical Pharmacology*.

[59] Murmu RP, Li W, Holtmaat A, Li J-Y (2013). Dendritic spine instability leads to progressive neocortical spine loss in a mouse model of huntington’s disease. *Journal of Neuroscience*.

